# Psoriasiform rash after initiation of treatment with olanzapine and carbamazepine.

**DOI:** 10.1192/j.eurpsy.2023.2148

**Published:** 2023-07-19

**Authors:** M. Abdelkefi, F. Guermazi, H. Trigui, R. Masmoudi, I. Baati, J. Masmoudi

**Affiliations:** psychiatry A, Hedi Chaker university hospital, sfax, Tunisia

## Abstract

**Introduction:**

olanzapine and carbamazepine are effectively used in treatment of schizoaffective disorder but they can have numerous side effects including skin eruptions that can be severe sometimes.

**Objectives:**

To study the relationship between toxidermia and treatment with Olanzapine and Carbamazepine.

**Methods:**

We report the case of a patient who developed a psoriasiform skin rash following the intake of Olanzapine and Carbamazepine.

**Results:**

Mrs SL, 46 years old, with no prior medical or surgical history, has been diagnosed with schizophrenia since the age of 26. She was initially treated with Haloperidol and Risperidone with an irregular follow-up. Then, due to the emergence of mood disorder symptoms, the patient was put on 20 mg/day of Olanzapine and 400 mg/day of carbamazepine. One month later, the patient presented a generalized rash requiring the discontinuation of the current medications. She was treated with corticosteroids, and then she was referred to our department to make the appropriate adjustment of her psychiatric treatment.

In view of the persistence of a dry erythroderma with erosive lesions of scratching and palmoplantar keratoderma, a skin biopsy was performed showing a psoriasiform and eosinophilic dermatosis that could be consistent with a toxidermia. The pharmacovigilance investigation concluded the incrimination of Olanzapine and carbamazepine in this symptomatology and recommended to avoid their prescription for this patient.

The need for a mood stabilizer presented us with a challenge, particularly in view of the potential risk of cross-toxicity. We opted for the reintroduction of Risperidone with strict monitoring of the skin condition.

**Conclusions:**

Each prescribed drug must be considered as potentially capable of causing cutaneous reactions as an adverse effect. Both the prescriber and the patient must be made aware of this phenomenon.

**Disclosure of Interest:**

None Declared

